# INTERGENERATIONAL ENGAGEMENT IN MEDICAL EDUCATION: THE PIVOTAL ROLE OF OLDER ADULT MENTORS

**DOI:** 10.1093/geroni/igae098.1633

**Published:** 2024-12-31

**Authors:** Jennifer Severance, Leland Waters, Emily Ihara

**Affiliations:** Chair; Co-Chair; Discussant

## Abstract

Medical education engaging older adults as mentors can broaden students’ perspectives about social, cultural, economic, and environmental factors influencing health; increase knowledge and skills in collaborative practice; and improve attitudes towards older patients. The Health Resources and Services Administration funded a national network of Geriatric Workforce Enhancement Programs (GWEP) to train health professions students in assessing and addressing the primary care needs of older adults. This symposium examines GWEP models of medical education involving older adults as mentors to promote person-centered, age-friendly, and dementia-friendly care. First, presenters from the University of Washington and University of Minnesota GWEPs will describe dementia education programs pairing medical students and individuals with memory loss. This is followed by a project at the Virginia Commonwealth University GWEP engaging older adults as mentors to medical student teams. Finally, presenters from the University of North Texas Health Science Center GWEP will describe strategies to expand and sustain an older adult mentorship program through volunteer engagement and interprofessional collaboration. Presenters will discuss partnerships to develop and sustain older adult mentorship programs as well as challenges and lessons learned. The symposium will conclude with an exploration of the input, feedback, and perspectives collected from older adults about their involvement in medical education as well as opportunities to adapt these intergenerational models to a local context.

